# The molecular principles of gene regulation by Polycomb repressive complexes

**DOI:** 10.1038/s41580-021-00398-y

**Published:** 2021-08-16

**Authors:** Neil P. Blackledge, Robert J. Klose

**Affiliations:** Department of Biochemistry, University of Oxford, Oxford, United Kingdom

## Abstract

Precise control of gene expression is fundamental to cell function and development. Although ultimately gene expression relies on DNA-binding transcription factors to guide the activity of the transcription machinery to genes, it has also become clear that chromatin and histone post-translational modification have fundamental roles in gene regulation. Polycomb repressive complexes represent a paradigm of chromatin-based gene regulation in animals. The Polycomb repressive system comprises two central protein complexes, Polycomb repressive complex 1 (PRC1) and PRC2, which are essential for normal gene regulation and development. Our early understanding of Polycomb function relied on studies in simple model organisms, but more recently it has become apparent that this system has expanded and diverged in mammals. Detailed studies are now uncovering the molecular mechanisms that enable mammalian PRC1 and PRC2 to identify their target sites in the genome, communicate through feedback mechanisms to create Polycomb chromatin domains, and control transcription to regulate gene expression. In this Review, we discuss and contextualise the emerging principles that define how this fascinating chromatin-based system regulates gene expression in mammals.

## Introduction

The development of multicellular organisms relies on cells and tissues establishing unique gene expression programmes. To achieve this, signalling pathways converge on DNA-binding transcription factors, which guide the binding and activity of RNA polymerase II (Pol II) at gene promoters^1^. However, through studying these mechanisms it has become apparent that chromatin, and histone post-translational modification, can also profoundly influence transcription and gene expression.

The importance of chromatin modifying complexes in controlling development is exemplified by the Polycomb repressive system. Polycomb genes were initially discovered in *Drosophila melanogaster*, where they are required for repression of homeotic (Hox) genes and thus for body plan specification^2^. Polycomb genes were subsequently shown to have mammalian orthologues that have important roles in controlling gene expression throughout development^3^.

Since these initial discoveries, biochemical analyses have shown that Polycomb proteins typically assemble into one of two large multi-protein complexes that post-translationally modify histones. Polycomb repressive complex 1 (PRC1) mono-ubiquitylates histone H2A at Lys119 (H2AK119ub1)^4,5^, whereas PRC2 mono-, di- and tri-methylates histone H3 at Lys27 (H3K27me1, H3K27me2 and H3K27me3, respectively)^6-9^. Importantly, PRC1 and PRC2 tend to spatially converge on the same sites in the genome to form Polycomb chromatin domains, which are uniquely enriched in H2AK119ub1, H3K27me3 and Polycomb complexes^10-14^. Polycomb chromatin domains are then thought to counteract transcription, though the mechanisms that enable this remain incompletely defined.

Although early genetic and biochemical investigations of Polycomb proteins were focussed on *D. melanogaster*, detailed studies over the past decade have revealed that the Polycomb system has expanded in mammals and that some key molecular principles that define its function have diverged from *D. melanogaster*. Based on these discoveries, we have gained a new understanding of how mammalian Polycomb complexes identify their target sites to form Polycomb chromatin domains and ultimately control gene expression. Several Reviews have recently discussed the general features and functions of Polycomb systems across phyla, and in mammalian development and disease^2,3,15–18^. In this Review we discuss and contextualise our emerging understanding of the molecular principles that enable the functions of mammalian Polycomb complexes in gene regulation. We first introduce the diverse complement of mammalian Polycomb repressive complexes and describe their enzymatic activities. We examine how Polycomb repressive complexes identify their target sites in the genome and communicate with each other to initiate the formation of Polycomb chromatin domains. We then discuss the regulation of Polycomb chromatin domain formation and ultimately how this is integrated with and regulates gene expression. Finally, we conclude by considering future avenues of research that will provide further mechanistic understanding of the principles of Polycomb biology in health and disease.

### The wide repertoire of mammalian PRCs

The rapid progress in understanding mammalian Polycomb biology has relied on detailed and systematic biochemical interrogation of Polycomb repressive complexes and their histone modifying activities. This work has shown that Polycomb complexes comprise catalytic cores, which bind auxiliary proteins to create distinct PRC1 and PRC2 assemblies. In this section we introduce the composition, structure and enzymatic activity of the different Polycomb complexes, as a prerequisite for examining how these complexes underpin the functions of mammalian Polycomb complexes in gene regulation.

#### Polycomb repressive complex 1 and its E3 ubiquitin ligase activity

**Figure 1:**
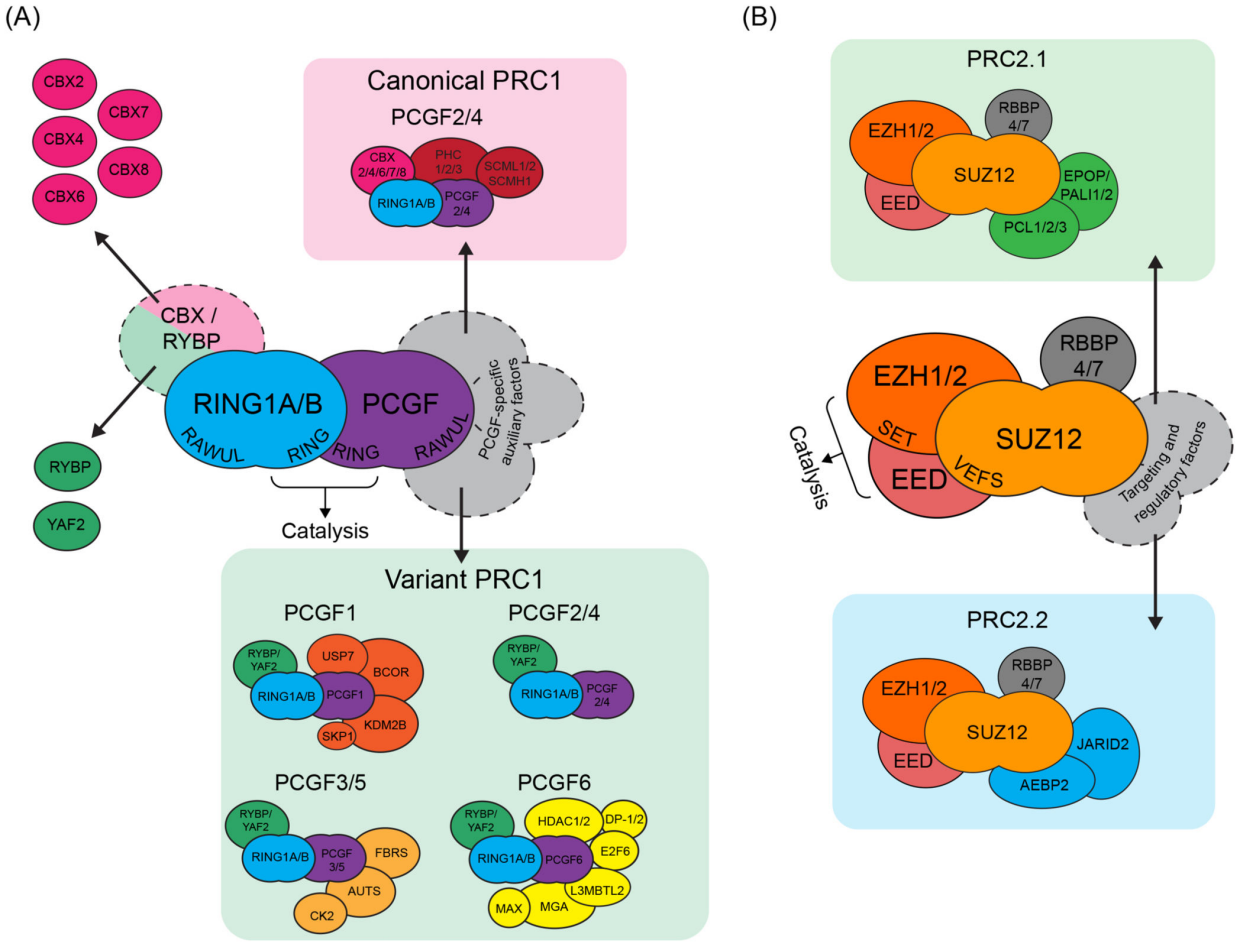
A diverse repertoire of mammalian Polycomb repressive complexes (A) The core of Polycomb repressive complex 1 (PRC1) comprises a RING1A or RING1B protein (RING1A/B) and one of six Polycomb group RING finger (PGCF) proteins (PCGF1–6). RING1 and PCGF proteins dimerise through their RING domains to form the catalytic core of PRC1, while the RING finger and WD40-associated ubiquitin-like (RAWUL) domains of both proteins interact with a range of auxiliary subunits, giving rise to biochemically distinct PRC1 complexes. Canonical PRC1 (cPRC1) complexes (top) assemble around PCGF2 or PCGF4, and include a chromobox protein (CBX2, 4, 6, 7 or 8) and Polyhomeotic (PHC) protein (PHC1, 2 or 3). In some cases, cPRC1 complexes also contain an SCM protein (SCML1 or 2 or SCMH1). By contrast, variant PRC1 (vPRC1) complexes (bottom) can assemble around all six PCGFs and contain RING and YY1 binding protein (RYBP) or YY1-associated factor 2 (YAF2). The identity of the PCGF protein dictates the incorporation of other auxiliary subunits, resulting in a number of distinct vPRC1 complexes. (B) The catalytic lobe of PRC2 is formed by a SET (Su(var)3-9, Enhancer-of-zeste and Trithorax)-domain containing enhancer of zeste 1 (EZH1) or EZH2 protein, together with embryonic ectoderm development (EED) and the VEFS (VRN2-EMF2-FIS2-SUZ12) domain of suppressor of zeste 12 (SUZ12). The N-terminal part of SUZ12 forms a distinct regulatory lobe that interacts with retinoblastoma-binding protein 4 (RBBP4) or RBBP7 and with other, auxiliary subunits that give rise to distinct PRC2.1 (top) and PRC2.2 (bottom) complexes. PRC2.1 complexes contain a Polycomblike (PCL) subunit (PCL1/2/3) and Elongin BC and Polycomb repressive complex 2-associated protein (EPOP) or PRC2-associated LCOR isoform 1 (PALI1) or PALI2, whereas PRC2.2 complexes contain adipocyte enhancer binding protein 2 (AEBP2) and Jumanji and AT-rich interaction domain containing 2 (JARID2). Protein domains are italicised. AUTS2, autism susceptibility protein 2; BCOR, BCL6 corepressor; CK2, casein kinase 2; DP-1, dimerization partner 1 (also known as transcription factor Dp-1); E2F6, transcription factor E2F6; FBRS, fibrosin; HDAC1, histone deacetylase 1; KDM2B, lysine-specific demethylase 2B; L3MBTL2, lethal(3)malignant brain tumour-like protein 2; MAX, MYC-associated factor X; MGA, MAX gene-associated; SCMH1, sex combs on midleg homolog 1; SCML1, SCM like 1; SKP1, S-phase kinase-associated protein 1; USP7, ubiquitin carboxyl-terminal hydrolase 7.

The catalytic core of PRC1 is composed of RING1B or its paralogue RING1A, and one of six Polycomb group ring finger (PCGF) proteins (PCGF1, 2, 3, 4, 5, or 6; Figure 1A)^19^. RING1 and PCGF proteins have similar domain architecture, including an N-terminal RING domain and C-terminal RING-finger and WD40-associated ubiquitin-like (RAWUL) domain. The RING1 protein and the PCGF protein dimerise through their RING domains, which facilitates their interaction with an E2 conjugating enzyme to enable histone ubiquitylation^20-22^. Their RAWUL domains bind to a range of auxiliary subunits, which regulate the catalytic activity of PRC1 and target it to specific sites in the genome^23,24^. Importantly, the PCGF components dictate which auxiliary subunits are incorporated into specific PRC1 complexes. These complexes are generally categorised as either canonical PRC1 (cPRC1) or variant PRC1 (vPRC1; Figure 1A), a nomenclature that reflects the order in which these complexes were initially identifed^19,23^. cPRC1 complexes, which were the first to be isolated^25,26^, assemble around either PCGF2 or PCGF4 and include one of five chromodomain-containing paralogues (CBX2, 4, 6, 7 or 8) and a Polyhomeotic (PHC) subunit (PHC1, 2 or 3). By contrast, vPRC1 complexes, which were identified later^19,27^, can assemble around any of the six PCGF proteins (PCGF1–6) and include RING1 and YY1-binding protein (RYBP), or its paralogue YAF2, and various additional subunits depending on the PCGF component present in the complex.

A key function of PRC1 complexes is to mono-ubiquitylate histone H2A. Given that E3 ubiquitin ligases are notoriously promiscuous, and that histones have a high density of potential Lys acceptor residues, it was initially puzzling how mammalian PRC1 could preferentially target Lys119 of H2A. The solution to this problem was provided by a landmark study detailing the atomic structure of a RING1B–PCGF4 RING domain dimer bound to an E2 ubiquitin conjugating enzyme and the nucleosome^28^. This structure revealed extensive contacts between PRC1 and the nucleosome acidic patch, which is a binding surface used by many nucleosome-interacting factors^29^. Furthermore, the E2 enzyme contacts the N-terminus of RING1B and DNA at the nucleosome dyad, thereby orienting itself to transfer ubiquitin to H2AK119 and explaining the substrate specificity of PRC1.

Although all six mammalian PCGF proteins have highly similar RING domains, the ubiquitin ligase activity of the PRC1 complexes they form differ considerably. Notably, vPRC1 complexes are much more active on nucleosome substrates than their cPRC1 counterparts^19,30,31^. Although these catalytic differences stem in part from the identity of the PCGF component directly influencing the activity of the core complex, incorporation of the vPRC1-specific auxiliary subunit RYBP also dramatically stimulates E3 ligase activity^19,30,32^. By contrast, incorporation of CBX proteins into cPRC1 complexes has a far less pronounced stimulatory effect and cPRC1 complexes are thus far less active than vPRC1 complexes.

#### Polycomb repressive complex 2 and its methyltransferase activity

PRC2 complexes form around a tetrameric core complex consisting of EZH2 or its paralogue EZH1, EED, SUZ12, and RBBP4 or RBBP7 (Figure 1B). Biochemical and structural analyses of PRC2 have largely focused on EZH2-containing complexes, which contribute more prominently to H3K27 methylation in most contexts and are essential for early development^33^. Cryo-EM structures have revealed that PRC2 forms two distinct lobes: a catalytic lobe and a targeting and regulatory lobe, which are bridged by SUZ12^34-36^. Within the catalytic lobe, EED and the VEFS (VRN2-EMF2-FIS2-SUZ12) domain of SUZ12 interact with EZH1 or EZH2, causing its SET (Su(var)3-9, Enhancer-of-zeste and Trithorax) domain to transition from an autoinhibitory conformation into an active one that supports methyltransferase activity^34,37-41^. Also important for the catalytic activity of PRC2 is the CXC domain of EZH2, which engages with nucleosomal DNA, and a patch of residues on the surface of EZH2, which interacts with the N-terminal portion of the H3 tail^42,43^. Together, these interactions position the N-terminal tail of H3 such that Lys27 is adjacent to the active site of PRC2, to enable its methylation.

The targeting and regulatory lobe of PRC2 includes the N-terminal portion of SUZ12, which binds to RBBP4 or RBBP7 and interacts with various additional auxiliary factors in a mutually exclusive manner^44^, giving rise to PRC2 assemblies with distinct biochemical properties (Figure 1B). PRC2.1 complexes contain a Polycomblike (PCL) protein (PCL1, 2, or 3) and either PRC2-associated LCOR isoform 1 (PALI1), PALI2 or Elongin BC and Polycomb repressive complex 2-associated protein (EPOP)^45-47^. By contrast, PRC2.2 complexes contain JARID2 and AEBP2. Importantly, PRC2.1-specific and PRC2.2-specific subunits also affect PRC2 methyltransferase activity^48^. For example, within PRC2.2, AEBP2 contains a patch of basic amino acids that contribute to nucleosome binding and stimulate H3K27 methylation^49^. Similarly, incorporation of either EPOP or PALI1 into PRC2.1 supports its methyltransferase activity through an undefined mechanism^47,50^. Interestingly, a protein that is predominantly expressed in germ-cells, EZH inhibitory protein (EZHIP), was recently shown to interact with the core PRC2 complex and inhibit its methyltransferase activity while also blocking interactions with PRC2.1-specific and PRC2.2-specific subunits^51-54^. This function suggests that context-specific regulators of PRC2 activity have important roles during development.

### Target site identification

Despite an intrinsic capacity to engage with nucleosomes, genome-wide studies have shown that Polycomb complexes are enriched at gene promoters and other gene regulatory elements^10,11,55-63^, indicating that more specific mechanisms must dictate the observed binding patterns. In the following sections, we examine emerging evidence that primary mechanisms of Polycomb complex targeting in mammals rely on a complement of sequence-specific DNA-binding factors, RNA-dependent mechanisms and binding to CpG islands (CGIs).

#### Target site recognition through sequence-specific DNA-binding factors

**Figure 2:**
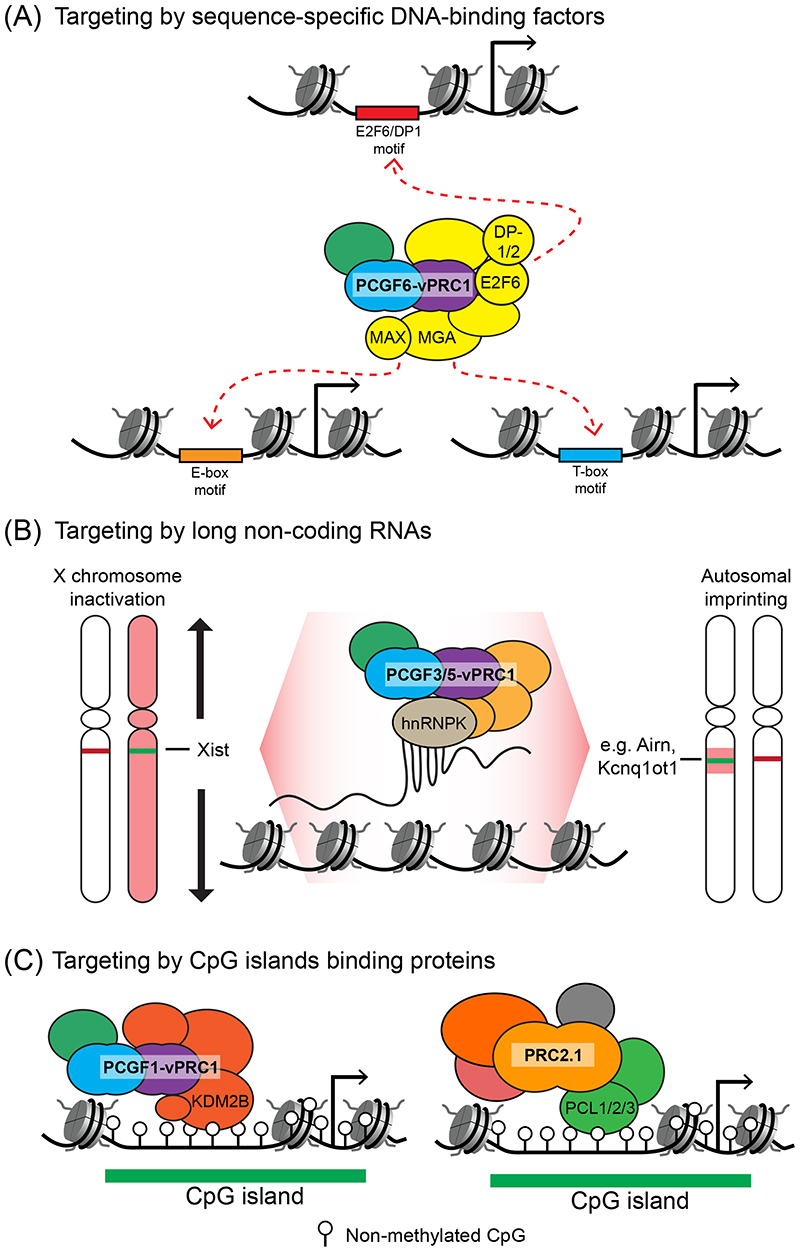
Primary mechanisms of Polycomb target-site identification (A) Sequence-specific DNA-binding factors in the Polycomb group RING finger 6 (PCGF6)-containing variant Polycomb repressive complex 1 (vPRC1) complex recognise DNA sequence motifs at target sites. These DNA-binding factors include MAX gene-associated (MGA)–MYC-associated factor X (MAX) and E2F6–dimerization partner 1 or 2 (DP-1/2) dimers, which bind to different DNA sequence motifs and contribute to sequence-specific PCGF6-vPRC1 targeting in different contexts. (B) Polycomb complexes can identify chromosomal binding sites during X chromosome inactivation through the long-noncoding RNA (lncRNA) X inactivation specific transcript (*XIST*); in autosomal imprinted regions mono-allelic gene repression is achieved through the lncRNAs *Airn* and *Kcnq1ot1*. In these regulatory contexts, the adaptor protein heterogeneous nuclear ribonucleoprotein K (hnRNPK) is thought to interact with the lncRNA and recruit the PCGF3/5-vPRC1 complex. These mono-allelic targeting cases are atypical in that they nucleate the binding of Polycomb complexes at large chromosomal regions, whereas Polycomb complex targeting to the majority of genomic sites is more punctate and associated with gene regulatory elements (see parts A and C). (C) Both PCGF1-vPRC1 and PRC2.1 are targeted to CpG islands. The lysine-specific demethylase 2B (KDM2B) subunit of PCGF1-vPRC1 contains a zinc finger-CxxC (ZF-CxxC) domain that binds specifically to non-methylated CpG dinucleotides. In PRC2.1, the Polycomb-like 1 (PCL1), PCL2 or PCL3 subunit contains a winged-helix domain that binds non-methylated CpG dinucleotides in certain sequence-contexts.

In *D. Melanogaster*, targeting of Polycomb complexes is thought to rely primarily on sequence-specific DNA-binding factors. However, many of these factors lack clear mammalian orthologues, or if they are present in mammals, do not target Polycomb complexes to chromatin^64-66^. This difference has brought into question whether these mechanisms are also important for mammalian Polycomb target site identification. In beginning to address this important question, it has recently been shown that the PCGF6-vPRC1 complex, which is absent in *D. Melanogaster*, stably incorporates auxiliary subunits that function as sequence-specific DNA-binding domains (Figure 2A)^19,23,67-69^. For example, the subunit MAX gene-associated protein (MGA) contains bHLH and T-box DNA-binding domains^70^. Studying these domains has revealed that MGA dimerises with MYC-associated factor X (MAX) to bind E-box motifs (5’-CACGTG-3’), and that it can also use its T-box domain to recognise longer T-box motifs (5’-TCACACCT-3’)^67,71-73^. These domains are important for PCGF6-vPRC1 occupancy at a subset of its target sites, which in somatic cells includes a number of germline-specific genes^67,74,75^. In addition, E2F6–DP-1 (or DP-2) heterodimers also associate with PCGF6-vPRC1 and provide the complex with distinct target-site specificity in other contexts^71,72^. Together, these findings demonstrate that PCGF6-vPRC1 utilises sequence-specific DNA-binding activities to identify a subset of its target sites in the genome (Figure 2A), and provide clear and direct evidence for sequence-specific targeting in mammals.

Other mammalian Polycomb repressive complexes do not stably incorporate proteins with sequence-specific DNA-binding domains. Nevertheless, certain Polycomb complexes can engage in more transient interactions with DNA-binding factors and this engagement appears to be important for target-site recognition in certain contexts. For example, PCGF3-vPRC1 recognises some of its target sites in embryonic stem cells (ESCs) by interacting with the transcription factors upstream stimulatory factor 1 (USF1) and USF2^72^. Other DNA-binding factors including REST, RUNX1 and SNAIL1 have been proposed to contribute to PRC1 or PRC2 targeting in certain cell types^76-79^. Despite these reports, in most cases direct interactions between DNA-binding factors and Polycomb complexes have not been unequivocally demonstrated, and they often only account for a subset of Polycomb complex binding events in a limited number of developmental contexts. Therefore, it appears unlikely that sequence-specific DNA-binding factors alone can explain how Polycomb complexes recognise their target sites in mammals.

#### Target site identification through chromatin-associated RNA

As an alternative to DNA-binding transcription factors, it was suggested that targeting of Polycomb complexes could be achieved through long non-coding RNA (lncRNAs) that associate with defined sites in chromatin^80^. Mammalian X chromosome inactivation (XCI), which is mediated by the lncRNA X inactive specific transcript (*XIST*), involves Polycomb repressive complexes. During XCI, *XIST* is expressed from one of the two female X chromosomes and spreads in *cis* to drive mono-allelic chromosomal gene silencing. Detailed characterization of this process has revealed that the PCGF3/5-vPRC1 complex interacts with the RNA-binding protein heterogeneous nuclear ribonucleoprotein K (hnRNPK), which recognises *XIST*, leading to PCGF3/5-vPRC1 enrichment on the inactive X chromosome (Figure 2B)^81-84^. However, this example is somewhat atypical for Polycomb complex targeting, in that *XIST* leads to enrichment of PCGF3/5-vPRC1 across the entire inactivated X chromosome, whereas on autosomes Polycomb complex enrichment is typically more restricted to gene regulatory elements.

Based on early observations made in *XIST* studies, numerous other lncRNAs have been proposed to target Polycomb complexes to sites in autosomes. Notably, the lncRNA HOX transcript antisense RNA (*HOTAIR*) was reported to function as a trans-acting repressor of the *HOXD* locus by physically interacting with and recruiting PRC2^85-87^. However, recent work has demonstrated that transcription repression by *HOTAIR* is independent of PRC2, and that PRC2 recruitment to the *HOXD* locus occurs primarily in response to gene silencing, not through direct targeting by *HOTAIR* ^88^. These findings highlight the need for caution when interpreting how lncRNAs influence gene expression and the recruitment of Polycomb complexes^80,89,90^. However, consistent with a potential role for lncRNAs in targeting the Polycomb complexes to chromatin, the lncRNAs *Airn* and *Kcnq1ot1* promote mono-allelic expression of autosomal genes in mouse trophoblast stem cells through a mechanism that appears to be highly analogous to XCI (Figure 2B)^91-93^. Like *XIST*, *Airn* and *Kcnq1ot1* associate with target sites in chromatin and utilise hnRNPK to form megabase-sized transcriptionally repressed genomic domains that are enriched for Polycomb complexes. In light of these findings, it is tempting to speculate that some lncRNAs have co-opted the Polycomb system as a mechanism to achieve mono-allelic gene regulation^94^.

In addition to lncRNAs, recent studies have suggested that RNA–DNA hybrid structures known as R-loops may also support Polycomb target-site recognition. R-loops were found to occur at a number of Polycomb target genes and their enzymatic resolution perturbed Polycomb complex targeting at some sites^95^. By contrast, in other contexts, R-loops were reported to negatively affect PRC2 binding and activity^96^. Although there appears to be a connection between Polycomb complexes and R-loops, the underlying biochemical mechanisms are unknown and the extent to which R-loops mediate Polycomb complex targeting to chromatin remains to be elucidated.

#### Targeting to CpG islands

Polycomb complexes appear to recognise some of their target sites using sequence-specific DNA-binding factors or through associating with lncRNAs. However, these mechanisms alone cannot explain all Polycomb complex binding observed in mammalian genomes, suggesting that additional targeting mechanisms exist. Early genome-wide studies revealed that Polycomb complexes associate primarily with CGIs^61,97^. CGIs are short (1–2 kb) regions of CpG-rich DNA that are associated with approximately 70% of mammalian gene promoters^98,99^. In CGIs, CpG dinucleotides remain free of DNA methylation, whereas elsewhere in the genome they are heavily methylated and function to maintain heterochromatin and repress the expression of parasitic DNA elements^100,101^. CGIs are absent in non-vertebrate model organisms, including *D. melanogaster*, which lacks DNA methylation. This led to speculation that CGIs may be used by Polycomb complexes to identify target gene promoters and other regulatory elements in mammals^61,102^.

Molecular evidence that CGI recognition may be important for Polycomb complex targeting first came from biochemical purifications of mammalian PRC1 complexes, in which the PCGF1-vPRC1 complex was shown to stably integrate lysine-specific demethylase 2B (KDM2B)^103,104^. KDM2B contains a zinc finger-CxxC domain that specifically binds to non-methylated CpG dinucleotides^105,106^. Consistent with this specificity, KDM2B can target the PCGF1-vPRC1 complex to CGIs (Figure 2C)^107-110^. PCL proteins in PRC2.1 complexes were also recently shown to bind to non-methylated CpG-containing DNA through a winged-helix domain that has a preference for certain sequences and is also influenced by the helical properties of DNA (Figure 2C)^111,112^. Consistent with *in vitro* evidence that PRC2 may function in some cases as a dimer^113^, it was recently proposed that a PRC2.1 dimer could stabilise binding to CpG-rich DNA, possibly by enabling simultaneous engagement with multiple non-methylated CpGs^114^. Although the precise contribution of dimerization to PRC2.1 function *in vivo* remains to be elucidated, the winged-helix domains of PCL proteins are crucial for PRC2.1 targeting, indicating that CGI binding is also central to target-site identification by PRC2^112^.

### The Polycomb chromatin domain

Once Polycomb complexes engage with target sites, this primary targeting must then be converted into the formation of repressive Polycomb chromatin domains, which can extend up to tens of kilobases and have extremely high levels of H2AK119ub1, H3K27me3, and Polycomb complex occupancy. In the following sections, we describe an intricate series of feedback and communication mechanisms that link the activity of Polycomb complexes on chromatin to enable the formation and maintenance of Polycomb chromatin domains.

#### H2AK119ub1 is recognised by PRC2.2 and vPRC1

**Figure 3:**
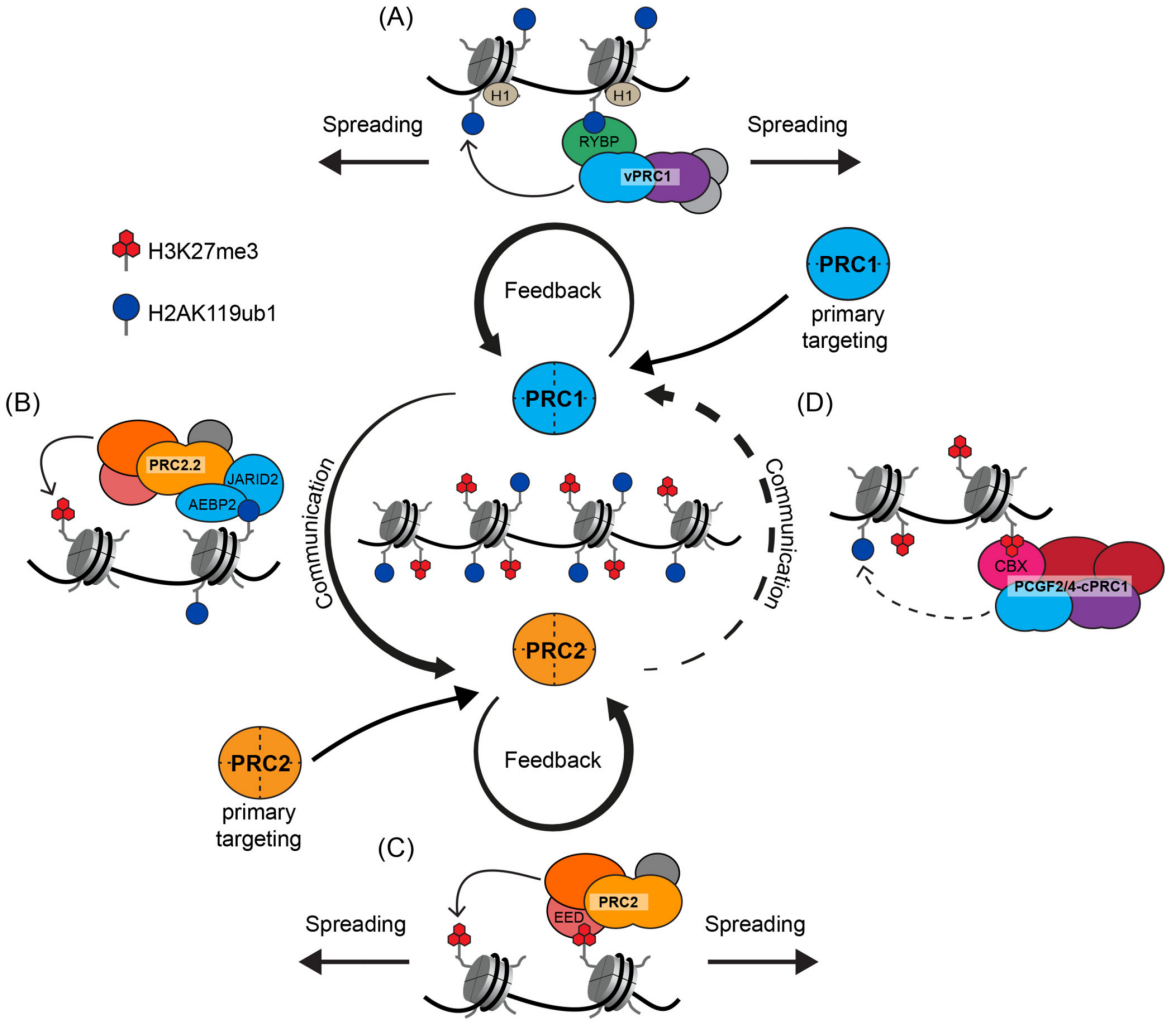
Formation and spreading of Polycomb chromatin domains Primary targeting of Polycomb repressive complex 1 (PRC1) and PRC2 is followed by feedback and communication mechanisms that enable the formation and spreading of Polycomb chromatin domains, which are characterised by elevated Polycomb complex occupancy and high levels of mono-ubiquitylated histone H2A Lys119 (H2AK119ub1) and tri-methylated histone H3 Lys27 (H3K27me3). (A) H2AK119ub1 is recognised by the RING and YY1 binding protein (RYBP) subunit (or by the YY1-associated factor 2 (YAF2) subunit; not shown) of variant PRC1 (vPRC1) complexes. This creates a feedback mechanism, supported by histone H1, that reinforces vPRC1 binding and amplifies H2AK119 ubiquitylation, thereby enabling spreading of vPRC1 and H2AK119ub1 away from the primary vPRC1 targeting site. (B) PRC1 complexes ubiquitylate H2AK119, which is recognised by the Jumanji and AT-rich interaction domain containing 2 (JARID2) and adipocyte enhancer binding protein 2 (AEBP2) subunits of PRC2.2. This causes elevated PRC2 occupancy and stimulates the tri-methylation of H3K27. Therefore, H2AK119ub1 facilitates communication between PRC1 and PRC2 in Polycomb chromatin domains. (C) H3K27me3 is recognised by the embryonic ectoderm development (EED) subunit of PRC2, which allosterically activates its methyltransferase activity. H3K27me3 creates a feedback mechanism that reinforces PRC2 binding and amplifies H3K27 tri-methylation, thereby enabling spreading of PRC2 and H3K27me3 away from the primary targeting site. (D) H3K27me3 is also recognised by the chromobox (CBX) subunit (CBX2, 4, 6, 7 or 8) of canonical PRC1 (cPRC1) complexes. Although cPRC1 complexes are less catalytically active than vPRC1 complexes (as indicated by the dotted arrow), in some contexts their activity may lead to H2AK119 ubiquitylation. Therefore, H3K27me3 can facilitate communication between PRC2 and PRC1 in Polycomb chromatin domains.

The complex regulation of mammalian gene promoters and the diversity of Polycomb targeting mechanisms have made it challenging to study the formation of Polycomb chromatin domains. To circumvent this difficulty, tethering experiments have been employed, in which specific Polycomb complexes can be artificially nucleated at an ectopic site in the genome and their capacity to communicate with other Polycomb components and to form Polycomb chromatin domains can be examined. Such experiments revealed that tethering of PRC1 was sufficient to drive *de novo* enrichment of PRC2, accumulation of H3K27me3 and the formation of a Polycomb chromatin domain that spread several kilobases from the primary engagement site^32,107,115^. Interestingly, this activity was exclusive to vPRC1 complexes and relied on their capacity to ubiquitylate H2AK119 (Ref.^30^) (Figure 3A). Subsequently, a biochemical link between H2AK119ub1 and PRC2 was narrowed down to the JARID2 subunit of PRC2.2, which can directly bind to H2AK119ub1 through a ubiquitin binding motif^116^; this interaction also stimulates the methyltransferase activity of PRC2^117^ (Figure 3B). The structural basis of this interaction was recently elucidated in a cryo-EM model of PRC2.2 bound to an H2AK119ub1-containing mono-nucleosome, which showed JARID2 interacts with mono-ubiquitylated H2A on one face of the nucleosome through its ubiquitin binding motif^118^. Interestingly, AEBP2 interacts with a second mono-ubiquitylated H2A on the opposite face of the nucleosome through tandem C2H2 zinc fingers. This indicates that auxiliary subunits in PRC2.2 have a specialised role in binding to H2AK119ub1-containing nucleosomes to tri-methylate H3K27, which is consistent with earlier biochemical work^116,117^. The importance of H2AK119ub1-mediated vPRC1 communication with PRC2 in the formation of Polycomb chromatin domains has also been shown through detailed genetic perturbation studies in mouse ESCs. Disruption of vPRC1 complexes caused major reductions in PRC2 occupancy and H3K27 tri-methylation in Polycomb chromatin domains, and this relied on their E3 ligase activity, consistent with ectopic tethering experiments and recent work in the developing mouse and zebrafish embryo^12,30,72,107,115,119-125^.

In addition to its influence on PRC2.2, H2AK119ub1 also reinforces chromatin binding and modification by vPRC1 through a zinc finger in RYBP, which can directly bind H2AK119ub1 (Figure 3A)^32,117,126^. This interaction forms the basis of a feedback mechanism that supports spreading of H2AK119ub1 onto neighbouring nucleosomes in a manner that appears to be aided by histone H1 (Ref.^32^). Together, these data demonstrate that once Polycomb target sites are identified through the primary targeting of vPRC1, feedback mechanisms inherent to vPRC1 complexes and H2AK119ub1 (Figure 3A), coupled with their capacity to communicate with PRC2.2 (Figure 3B), are essential for the formation and spreading of Polycomb chromatin domains.

#### H3K27me3 stimulates PRC2 activity and is recognised by cPRC1

Although H3K27me3 and the occupancy of PRC2.2 in Polycomb chromatin domains relies on PRC1 and H2AK119ub1, primary targeting modalities also enable PRC2 to identify target sites and catalyse H3K27 tri-methylation. PRC2 can then bind the product of its own catalysis, H3K27me3, through a WD40-repeat domain in EED, causing allosteric activation of its methyltransferase activity^39,127,128^ (Figure 3C). PRC2 activation creates a feedback mechanism that enables spreading of H3K27me3 along chromatin^129^. A recently elucidated cryo-EM model of PRC2 bound to a dinucleosome revealed that EED engages with one H3K27me3-containing nucleosome and positions the SET domain of EZH2 to methylate an unmodified H3 tail on an adjacent nucleosome ^43^. The geometry of this interaction also revealed that a short length of DNA linker between nucleosomes is important for optimal methyltransferase activity, which is consistent with evidence that PRC2 prefers more compact chromatin substrates^130^.

H3K27me3 is also recognised by a chromodomain in the CBX subunit of cPRC1 complexes, suggesting that PRC2 can communicate with cPRC1 through H3K27me3 (Figure 3D)^6,8,131,132^. In agreement with this possibility, early functional experiments showed that PRC2 activity was required for cPRC1 occupancy in Polycomb chromatin domains^10^. However, consistent with the fact that cPRC1 complexes have extremely low E3 ligase activity *in vitro* ^30^, PRC2 and cPRC1 contribute only minimally to H2AK119ub1 levels *in vivo* ^12,27,125,133^. Furthermore, ectopic tethering of PRC2 or cPRC1 to chromatin in ESCs did not lead to elevated H2AK119ub1 levels, indicating that these complexes are incapable of efficiently driving *de novo* Polycomb chromatin domain formation in this context^107^. Therefore, in contrast to vPRC1 complexes, which communicate effectively with PRC2.2 to drive H3K27 tri-methylation, PRC2 appears to work much less efficiently through cPRC1 to reinforce H2AK119 ubiquitylation. Nevertheless, overexpression of the cPRC1 subunit CBX7 was recently shown to support H3K27me3-dependent maintenance of H2AK119ub1 at an artificial target gene promoter^134^, suggesting that in some circumstances PRC2 may support this process more efficiently. In summary, while primary targeting modalities and recognition of H2AK119ub1 are important for nucleation of PRC2 at target sites, H3K27me3 deposition is necessary to reinforce PRC2 binding, support spreading of PRC2 and H3K27me3, and stabilise cPRC1 occupancy during Polycomb chromatin domain formation.

#### cPRC1 supports the organization of Polycomb chromatin domains in the nucleus

**Figure 4:**
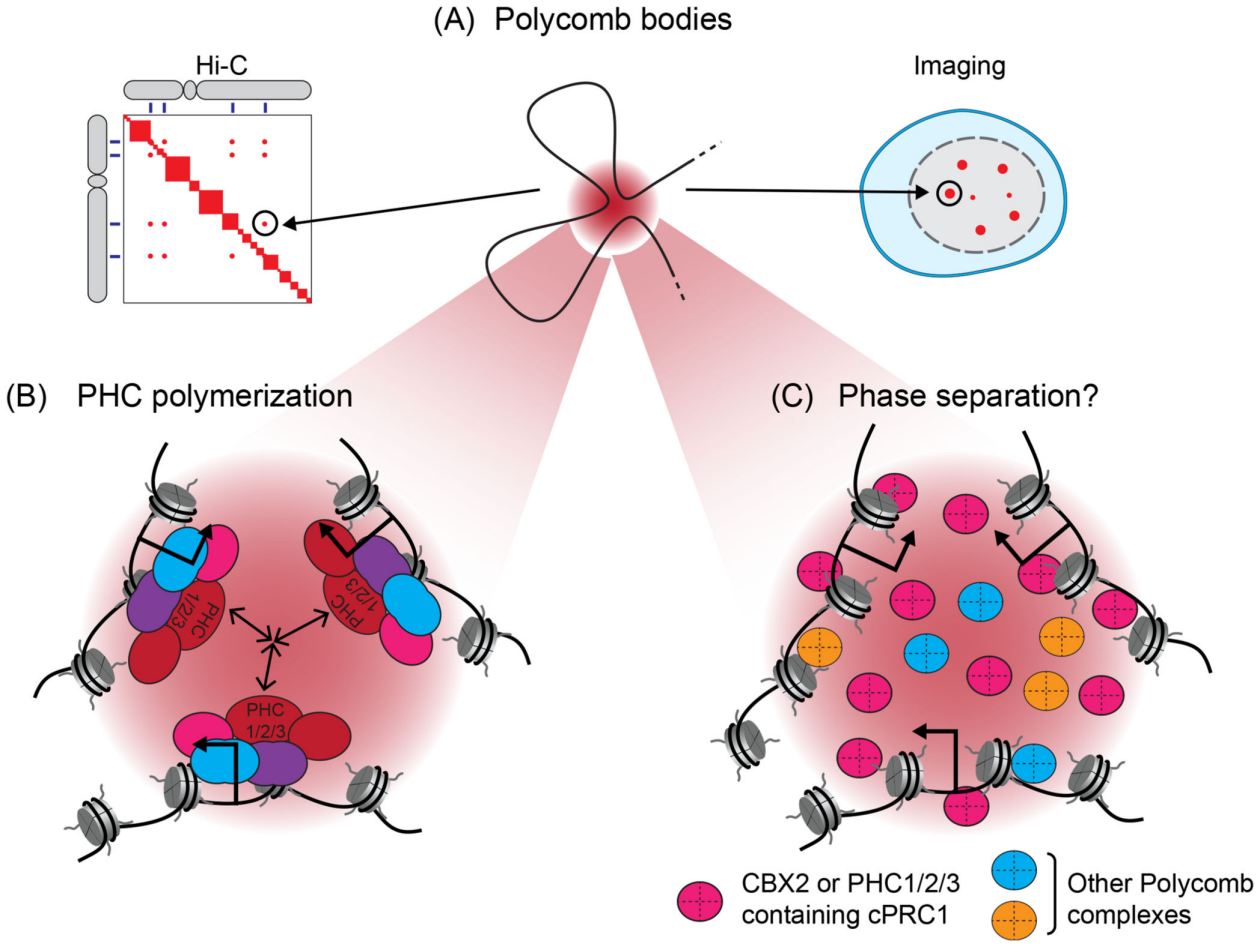
Polycomb bodies, long-range interactions, and phase separation. (A) Distinct Polycomb chromatin domains can interact in three dimensional space, even when separated by very large distances across the genome. In high-throughput chromosome conformation capture (Hi-C), these interactions correspond to regions of high contact frequency (left). In imaging experiments, they correspond to foci of Polycomb complex components, mono-ubiquitylated histone H2A Lys119 (H2AK119ub1) and tri-methylated histone H3 Lys27 (H3K27me3), which are often referred to as Polycomb bodies (right). (B) Long-range interactions between Polycomb chromatin domains require the Polyhomeotic (PHC) subunit (PHC1, 2 or 3) of canonical Polycomb repressive complex 1 (cPRC1), which can polymerise through its sterile alpha motif (SAM). (C) Components of cPRC1, including chromobox 2 (CBX2) and the SAM domain of PHC1, 2 or 3, can undergo liquid–liquid phase separation *in vitro* and form nuclear condensates *in vivo*, which share similarities with Polycomb bodies. These condensates could possibly augment Polycomb complex activities and may reinforce long-range interactions between Polycomb chromatin domains.

Although cPRC1 complexes contribute minimally to H2AK119 ubiquitylation, they have evolved additional biochemical features that enable them to control the spatial organisation of Polycomb chromatin domains in the nucleus. Imaging and chromatin conformation capture-based approaches have demonstrated that even when separated by up to several megabases across a chromosome, distinct Polycomb chromatin domains can interact in three-dimensional space^135-142^ (Figure 4A). In imaging experiments, these interactions appear as focal accumulations of Polycomb proteins, which are often referred to as Polycomb bodies^143-145^. The formation of these interactions relies on PHC proteins in cPRC1 complexes, which at least *in vitro* can utilise their sterile alpha motif (SAM) to polymerise in a head-to-tail orientation and form long filaments^146-150^ (Figure 4B). How these filaments support three-dimensional chromatin interactions remains poorly understood, but one could envisage cPRC1 filaments bridging distinct Polycomb chromatin domains. Although Polycomb chromatin domains form prominent interactions in three-dimensional space, they are not static structural entities^151^. Instead, binding of Polycomb proteins at these sites is highly dynamic^144,152-155^ and cohesin, which dynamically extrudes chromatin loops, counteracts interactions between Polycomb chromatin domain^139,156,157^.

In addition to supporting long-range interactions between Polycomb chromatin domains, components of cPRC1 complexes can undergo liquid–liquid phase separation *in vitro* and form nuclear condensates *in vivo*, which appear similar to Polycomb bodies^158,159^ (Figure 4C). The capacity for liquid–liquid phase separation resides with CBX2, which contains a positively charged disordered region, and with the SAM of PHC proteins through a mechanism that is enhanced by, but not strictly dependent upon, the capacity of PHC to polymerise ^158-161^. Condensates can enhance cellular processes by sequestering or excluding macromolecules^162^. In line with this concept, condensates formed by CBX2 or the PHC SAM domain were shown to concentrate nucleosomes and nucleic acids, while also permitting entry of other Polycomb complexes, suggesting that condensates could augment Polycomb activities on chromatin^161^ or limit the access of other factors to Polycomb chromatin domains. However, quantitative imaging has also shown that concentrations of cPRC1 components inside Polycomb bodies are in some contexts below what is thought to be required to support liquid–liquid phase separation, bringing into question whether Polycomb bodies are formed by this process^152,163^. Further study of Polycomb protein condensates and their relevance to Polycomb functions is therefore required.

#### The maintenance of Polycomb chromatin domains following DNA replication

The integrity of Polycomb chromatin domains is challenged by passage of the replication machinery which displaces parental nucleosomes from DNA and necessitates the incorporation of new unmodified histones and the reincorporation of modified parental histones into the replicated daughter strands^164,165^. Communication and feedback mechanisms inherent to Polycomb complexes^32,127^ could in theory support copying and epigenetic maintenance of Polycomb chromatin domains, but this would rely on modified parental nucleosomes being deposited in the same location on daughter strands. Elegant experiments examining the kinetics of histone deposition during DNA replication have recently shown that H3K27me3-containing nucleosomes are incorporated into daughter DNA strands almost precisely where they originated from in the parental DNA ^166,167^, providing a potential conduit for maintaining the identity of Polycomb chromatin domains^168,169^.

Interestingly, however, a considerable lag exists between DNA replication and the establishment of H3K27me3 on the newly incorporated, unmodified histone H3, despite reports that Polycomb repressive complexes can travel with the replication machinery^169-171^. Furthermore, H3K27me3 profiles can be re-established *de novo* following complete erasure of H3K27me3^129,172^ and work in *D. melanogaster* has shown that long-term maintenance of H3K27me3 in Polycomb chromatin domains relies on the underlying DNA sequence^173,174^. Together, these observations have brought into question the extent to which epigenetic maintenance through feedback mechanisms in Polycomb chromatin domains contributes to their re-establishment following DNA replication. It will be important to understand in more detail the kinetics of re-establishment of Polycomb chromatin domains following replication, including whether parental H2AK119ub1 is also faithfully redeposited. Understanding these basic principles will be essential for defining whether epigenetic maintenance is important for the function of Polycomb chromatin domains in actively dividing cells.

### Controlling Polycomb chromatin domains

**Figure 5:**
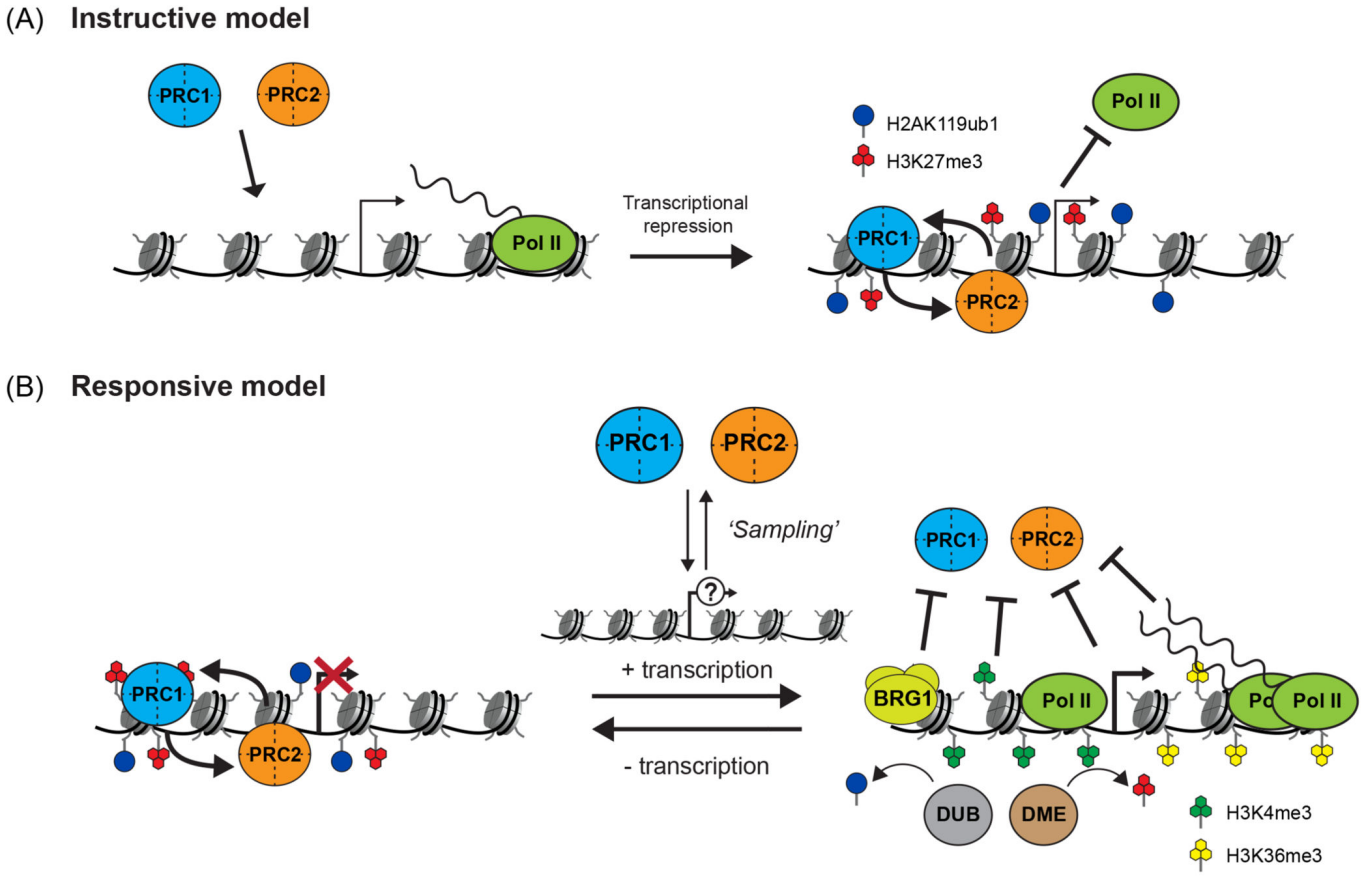
Models of Polycomb chromatin domain formation and gene regulation (A) An instructive model, in which Polycomb complexes are recruited to target sites (left), where they initiate the formation of Polycomb chromatin domains (right) and drive the repression of transcribed genes. (B) A responsive model, in which Polycomb complexes dynamically ‘sample’ potential target sites for susceptibility to Polycomb chromatin domain formation. In particular, the Polycomb group RING finger 1 (PCGF1)-containing variant Polycomb repressive complex 1 (vPRC1) and PRC2.2 complex, through their capacity to bind CpG islands (not shown), could dynamically engage with approximately 70% of gene promoters. At lowly or untranscribed genes (left), these complexes could potentially sense and respond to the (near) absence of transcription by ubiquitylating histone H2A Lys119 (H2AK119ub1) and tri-methylating histone H3 Lys27 (H3K27me3) to initiate the formation and spreading of Polycomb chromatin domains, which could help counteract low-level or inappropriate transcription and maintain an inactive chromatin state to protect cell identity. However, at expressed genes (right), transcription-associated features including H3K4me3 and H3K36me3, high levels of nascent transcripts, BRG1-mediated chromatin remodelling, and deubiquitylase (DUB) and demethylase (DME) activities, counteract H2AK119 and H3K27 modification, thereby blocking Polycomb chromatin domain formation and limiting Polycomb function at these genes. Pol II, RNA polymerase II.

It is often posited that chromatin modifying complexes are recruited to defined target sites in the genome, where they modify histones to drive gene activation or repression^175^. However, these simple instructive models appear for the most part to be incompatible with our evolving understanding of where and how Polycomb chromatin domains form and function. If instructive processes dictated Polycomb chromatin domain formation (Figure 5A), one would expect that all sites in the genome where primary targeting mechanisms allow Polycomb complexes to engage would acquire repressive Polycomb chromatin domains. However, genome-wide studies have shown that only a small subset of sites where Polycomb complex binding can be detected ultimately go on to form Polycomb chromatin domains that have high levels of H2AK119ub1, H3K27me3 and Polycomb complex occupancy^12,133,176,177^. For example, the PCGF1-vPRC1 complex is detected at most CGIs in the genome, albeit at low levels^12,108-110^. Yet, despite the inherent *de novo* Polycomb chromatin domain-forming activity of PCGF1-vPRC1^107^, only ~20% of CGIs in a given cell type will acquire a domain^108,112^, indicating that this process must be highly regulated. In this section we discuss the emerging principles that regulate Polycomb chromatin domain formation and how this is related to transcription.

#### Transcription regulates susceptibility to Polycomb chromatin domain formation

A unifying feature of target sites that form Polycomb chromatin domains is extremely low or non-detectable levels of transcription, raising the possibility that transcription itself limits the activity of Polycomb complexes. In agreement with this notion, treating cells with transcription inhibitors can stabilise PRC2 binding and increase H3K27me3 levels at thousands of previously active CGI-associated gene promoters^178^. When a polyadenylation signal, which promotes transcription termination, was inserted adjacent to an active CGI-associated promoter, this had a similar effect^179^. These findings are consistent with experiments showing that synthetic CpG-rich non-methylated DNA can recruit Polycomb complexes and lead to the apparent formation of Polycomb chromatin domains, but only when these sites lack binding of transcription activators or are not in close proximity to enhancer elements^56,180,181^. Furthermore, experiments examining the kinetics of Polycomb chromatin domain formation during cellular-state transitions demonstrated that this primarily occurs following transcription cessation^130,182^. Together, these observations indicate that despite Polycomb complexes broadly engaging with regulatory elements via targeting modalities such as CGI recognition, transcription potently counteracts the formation of Polycomb chromatin domains.

#### Nascent RNA regulates the activity of Polycomb repressive complex 2

The realisation that gene activity appears to be incompatible with Polycomb chromatin domain formation has led to a concerted effort to understand the mechanisms that regulate this process. Although some lncRNAs appear to target Polycomb complexes to specific sites, an overwhelming body of evidence now also indicates that PRC2 binds promiscuously to nascent RNA^113,183-189^. Several studies have reported that nascent RNA competes for PRC2 binding and displaces it from chromatin, while also inhibiting its methyltransferase activity^179,190-193^, both of which would antagonise the formation of Polycomb chromatin domains at actively transcribed sites. However, other studies have contradicted this view and proposed that interaction with nascent RNA, either directly by PRC2 (Ref.^194^) or through the pre-mRNA splicing factor RBFOX2 (Ref.^195^), supports PRC2 occupancy at target sites.

To reconcile these seemingly contradictory effects, an ‘RNA-bridging model’ has been proposed^194^. In this model, nascent transcripts from very lowly transcribed genes help bring PRC2 into proximity with promoter elements to support chromatin binding and tri-methylation of H3K27. However, at more highly transcribed genes, elevated levels of nascent transcripts would compete with PRC2 for chromatin binding and decrease H3K27 tri-methylation. Although the molecular details of this model remain to be fully tested, it appears that PRC2 is highly attuned to sensing transcript levels and using this information to regulate the formation of Polycomb chromatin domains.

#### The influence of transcription-associated chromatin features

In addition to producing nascent RNA, transcription also profoundly influences the chromatin environment at transcribed genes. For example, during transcription initiation, histone methyltransferases tri-methylate H3K4 at gene promoters, whereas elongation is associated with H3K36 tri-methylation in gene bodies^97,196^. Importantly, *in vitro* studies have demonstrated that the methyltransferase activity of PRC2 is inhibited by the presence of H3K4me3, H3K36me2 or H3K36me3 (Ref.^42,197-202^), and disruption of H3K4 methyltransferase activity *in vivo* can lead to enhanced Polycomb chromatin domain formation^203^. In addition, histone demethylases act as part of transcription co-activator complexes to remove H3K27 methylation^204-209^, and transcription-associated H3K27, H4K16 and H4K20 acetylation counteracts PRC2 activity^210-215^. Similarly, the H2AK119ub1-specific deubiquitylases USP16 and 2A-DUB (also known as MYSM1) have been shown to associate with promoters of actively transcribed genes and counteract H2AK119 ubiquitylation^216,217^.

In addition to histone modification-dependent effects, the chromatin remodelling ATPase BRG1 can actively evict PRC1 from promoters of transcribed target genes^218-220^. This activity was reported to target RYBP-containing vPRC1 complexes, suggesting that promoter-associated chromatin remodelling activities can displace vPRC1 complexes and limit H2AK119 ubiquitylation^221^. Together, these findings suggest that although primary targeting activities may enable Polycomb complexes to engage with a wide range of gene promoters, transcription-associated histone modifications and chromatin remodelling activities at regulatory elements can limit the catalysis and feedback mechanisms required for efficient formation of Polycomb chromatin domains.

#### Target sampling and a responsive model of Polycomb chromatin domain formation

Our growing understanding of Polycomb complex targeting and the mechanisms that control Polycomb chromatin domain formation appear to be consistent with a dynamic sampling mode of action that is responsive to transcription^222^ (Figure 5B). According to this model, DNA-binding activities associated with Polycomb complexes would provide a mechanism to dynamically engage with, or sample, potential target sites. Central to this process would be PCGF1-vPRC1 and PRC2.1, which can recognise non-methylated DNA in CGI-elements that are associated with most mammalian gene promoters^108-112^. Sampling could allow Polycomb complexes to constantly interrogate a wide range of potential target sites, with only those that are lowly or not transcribed being susceptible to Polycomb-mediated histone modification, which is necessary to initiate the feedback and communication mechanisms required for efficient formation and spreading of Polycomb chromatin domains.

A responsive mode of Polycomb function based on target site sampling would in theory provide a generic mechanism to form Polycomb chromatin domains at non- or lowly transcribed genes without the need to evolve complex cell-type specific targeting mechanisms. As such, the Polycomb system could function to protect inactive genes against low-level or inappropriate transcription activation signals and help to maintain gene repression in many, if not all, cell types. This model is also consistent with early genetic experiments in fruit flies and mammals, in which Polycomb proteins were observed to maintain, rather than initiate, gene repression^3,223-225^. Nevertheless, more work is required to test the validity of a sampling-based model for the formation and function of Polycomb chromatin domains in different biological contexts.

### Polycomb-mediated gene regulation

After receptive target sites establish Polycomb chromatin domains, these then have an important role in controlling gene expression. In this section we examine the mechanisms of Polycomb chromatin domain-mediated gene repression, discuss atypical cases of gene activation by Polycomb complexes, and consider how CGIs might utilise Polycomb complexes to create bistable chromatin states that shape gene expression during cell differentiation.

#### Transcription repression by Polycomb chromatin domains

Early models posited that the Polycomb system may repress transcription through cPRC1 binding to H3K27me3 and compacting chromatin to limit access to gene regulatory elements^137,149,150,158,159,226,227^. Although this mechanism may contribute to Polycomb-mediated gene repression in some contexts, mice deficient for cPRC1 components often have milder phenotypes than mice in which vPRC1 components are perturbed, and cPRC1-deficient ESCs exhibit relatively few gene expression changes^12,67,81,107,148,225,228-230^. Furthermore, chromatin accessibility does not always change when Polycomb systems are disrupted^231,232^. Together, these findings indicate that, at least during early development and in ESCs, Polycomb chromatin domain features distinct from H3K27me3 and cPRC1 must contribute centrally to transcription repression.

**Figure 6:**
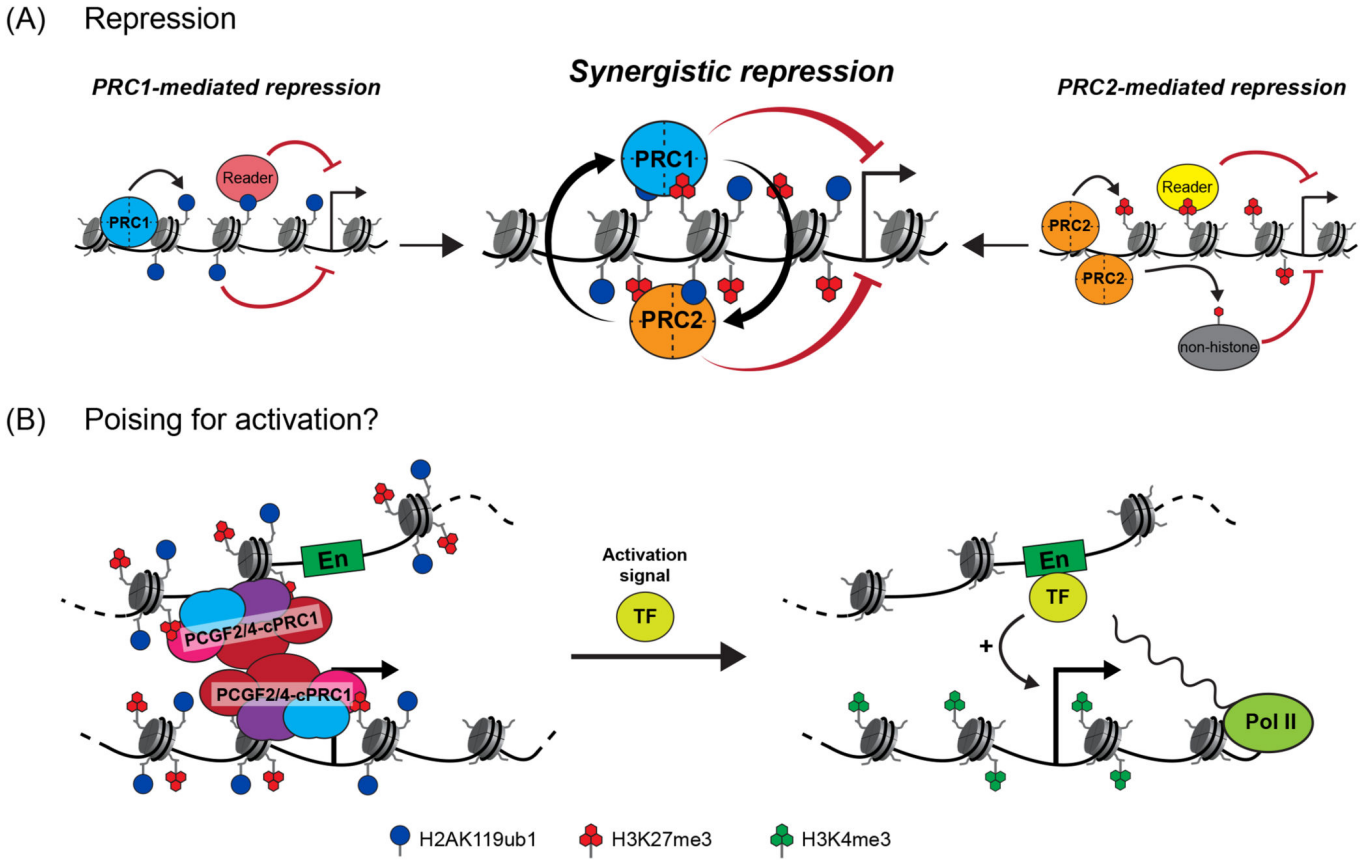
Mechanisms of Polycomb-mediated gene regulation (A) Despite the integration of their activities in Polycomb chromatin domains, the mechanisms that enable Polycomb repressive complex 1 (PRC1) and PRC2 to counteract transcription appear to be distinct. PRC1-mediated gene repression is driven by ubiquitylation of histone H2A Lys119 (H2AK119ub1), possibly mediated by the activity of H2AK119ub1-reader proteins or, more directly, by the installation of the bulky ubiquitin moiety into chromatin and thus antagonising some aspect of transcription (left). PRC2-mediated repression appears to involve readers of tri-methylated histone H3 Lys27 (H3K27me3) or methylation of non-histone substrates (right). Importantly, although PRC1 and PRC2 can independently counteract transcription, the communication and feedback between PRC1 and PRC2, which support the formation of Polycomb chromatin domains, appear in some contexts to synergise the repressive activities of PRC1 and PRC2 at target genes (centre), thereby providing a robust barrier against inappropriate gene expression. (B) In some contexts, Polycomb complexes can activate genes. Canonical PRC1 (cPRC1) mediates the formation of chromatin topologies that can bring poised enhancers (En) and their target promoters into close proximity (left). Once activation signals are received at the enhancer through transcription factor (TF) binding, this could support rapid induction of transcription (right). PCGF2/4, Polycomb group ring finger 2 or 4; Pol II, RNA polymerase II.

In line with this possibility, recent studies have pointed towards a central role for H2AK119ub1 and vPRC1 in gene repression. This is evident in ESCs, where disruption of PRC1 resulted in increased expression of thousands of Polycomb target genes, effects which were almost entirely dependent on PCGF1/3/5/6-vPRC1 complexes^12^. Importantly, disruption of the catalytic activity of PRC1 recapitulated these gene expression defects despite vPRC1 binding being retained at target sites, suggesting that gene repression by PRC1 in this context is mediated by H2AK119 ubiquitylation^119,121^. H2AK119ub1 having a central role in gene repression is consistent with PRC1 binding to chromatin being highly dynamic and displaying extremely low target site occupancy^152,154^. Although the precise mechanism(s) by which H2AK119ub1 affects transcription remain to be determined (Figure 6A), it does not appear to rely on PRC2 occupancy^233^. Furthermore, although H2AK119ub1 reader proteins, including RYBP and the chromatin remodelling factor RSF1, have been linked with transcription repression^30,32,126,234^, their activities appear insufficient to account for PRC1-mediated gene repression. An alternative possibility is that the addition of a bulky ubiquitin moiety to H2A could more directly counteract the function of the transcription machinery^235,236^. Consistent with this possibility, acute disruption of PRC1 and loss of H2AK119ub1 caused rapid new binding of Pol II and elevated the frequency of transcription bursts, suggesting PRC1 and H2AK119ub1 affect transcription initiation^233^, which is in agreement with previous reports that PRC1 might antagonise the assembly or activity of the transcription pre-initiation complex^237,238^. Other reports have suggested that PRC1 limits transcription by constraining a post-initiation, promoter-proximal poised form of Pol II^239,240^. More detailed biochemical work is required to define the mechanisms by which H2AK119ub1 influences transcription.

Consistent with the catalytic activity of PRC1 being required for Polycomb-mediated gene repression in ESCs, an essential role for H2AK119ub1 in Polycomb target gene repression was recently demonstrated by examining the early stages of neuronal cell fate restriction^241^. However, this requirement was less pronounced at later stages of neuronal differentiation, when a shift towards H2AK119ub1-independent gene repression was observed. These findings suggest that H2AK119ub1 could possibly confer a form of repression in stem cells that can be overcome in response to appropriate developmental gene expression cues. By contrast, as cells commit to particular lineages and subsets of genes will no longer be expressed, the Polycomb system may employ additional mechanisms, perhaps involving structural changes to chromatin, that enable long-term and robust maintenance of repression. This possibility has been supported by studies examining the contribution of the catalytic activity of PRC1 to mouse development, which reported that PRC1 uses a combination of H2AK119ub1-dependent and H2AK119ub1-independent mechanisms to control gene expression^119,123,133,137,241^. However, it has subsequently been shown that PRC1 catalytic mutations used in these studies do not fully eliminate H2AK119 ubiquitylation^119,241^. Nevertheless, in some cases in *D. melanogaster*, the repression of *Hox* genes and other Polycomb target genes can be maintained by PRC1 independently of H2AK119 ubiquitylation^242^. Clearly more work is required to define the contribution H2AK119ub1-dependent and H2AK119ub1-independent mechanisms to Polycomb-mediated gene repression in different cell types and developmental stages.

PRC2 also has important roles in Polycomb-mediated gene repression, yet its disruption in mammals typically has less severe effects on gene expression and development than removal of PRC1^10,124,125,243-248^. Studies in *D. melanogaster* have suggested that PRC2-mediated repression relies on H3K27 methylation, as a histone H3 Lys27-substitution mutation that renders this residue refractory to methylation largely phenocopies the gene expression and developmental defects observed when PRC2 is disrupted^249^. Conversely, a hyperactive form of EZH2, which causes increased levels of H3K27me3, drives inappropriate repression of target genes^250^. Consistent with these findings, chemical inhibitors that limit the methyltransferase activity of PRC2 and cause a reduction in H3K27 methylation, are associated with derepression of Polycomb target genes in mammals^251,252^, as is replication-mediated dilution of H3K27me3 (Ref.^252^). Recently, an evolutionarily-conserved bromo adjacent homology (BAH) domain in BAHD1 and BAHCC1 was shown to specifically recognise H3K27me3 and contribute to gene repression, possibly by recruiting histone deacetylases, which are known to counteract gene expression^253-255^ (Figure 6A). PRC2 also methylates non-histone substrates, including the transcription factors GATA4 and Elongin A, possibly as an alternative mechanism of counteracting transcription^256,257^.

#### PRC1 and PRC2 collaborate in Polycomb chromatin domains to repress transcription

As we understand more about the mechanisms used by Polycomb complexes to counteract transcription, it is becoming increasingly clear that PRC1 and PRC2 have independent gene repression activities, despite being linked by feedback mechanisms in Polycomb chromatin domains^258^. For example, in mouse ESCs or the developing epidermis, disruption of either PRC1 or PRC2 caused increased expression of a largely overlapping set of target genes^231,259,260^. However, removal of both PRC1 and PRC2 together caused a more pronounced derepression of target genes, and in the case of the developing epidermis, catastrophic morphological defects. These observations are consistent with the idea that the convergence of Polycomb complexes at target sites provides an opportunity for feedback mechanisms to create more expansive Polycomb chromatin domains in which the independent repressive activities of PRC1 and PRC2 can synergise to create a robust barrier against inappropriate transcription (Figure 6A). These properties may be particularly well suited to protecting gene expression programmes over developmental timescales, where the Polycomb system is known to have important roles^10,133,241,243-245,261^. The abundance and composition of individual Polycomb repressive complexes can change considerably over development^33,176,262-265^. This feature may provide an opportunity to regulate the repressive nature of Polycomb chromatin domains in different cellular and developmental contexts, for example by altering the relative contribution of PRC1-dependent and PRC2-dependent repression mechanisms.

#### Pervasive effects of Polycomb-mediated histone modification on gene expression

Although H2AK119ub1 and H3K27me3 levels are highly elevated in Polycomb chromatin domains, they are also found elsewhere in the genome, and there is mounting evidence that these alternative pools of H2AK119ub1 and H3K27me can affect gene expression in various contexts. The PCGF3/5-vPRC1 complexes deposit a ‘blanket’ of H2AK119ub1 across the genome, with approximately 10% of H2A molecules being modified ^12,266^. The extent of this pervasive pool of H2AK119ub1 is suppressed by the deubiquitylase BAP1^267,268^, the absence of which elevates H2AK119ub1 levels and constrains the expression of thousands of genes that are not normally influenced by PRC1 (Ref.^269-271^). This suggests that pervasive H2AK119 ubiquitylation may function like a rheostat to control the transcriptional potential of the genome.

PRC2 also acts pervasively throughout the genome: approximately 70% of H3K27 residues undergo mono-methylation or di-methylation and up to 15% undergo tri-methylation^272-275^. Although the mechanisms that control H3K27 methylation outside of Polycomb chromatin domains remain poorly understood, this pervasive activity primarily relies on the core complex and not on PRC2.1-specific and PRC2.2-specific subunits^274^. Importantly, there is evidence that these alternative pools of H3K27me can broadly antagonise transcription, possibly by counteracting H3K27 acetylation and limiting the activity of enhancers^272,276^. Therefore, it is clear that Polycomb complexes can also influence gene expression by functioning outside of Polycomb chromatin domains and it has recently been suggested that these activities may be particularly relevant in germ cells and in very early developmental stages-^124,125,277,278^.

#### Gene activation by Polycomb complexes

Although Polycomb complexes appear to primarily support transcription repression, there is increasing evidence that in some contexts, Polycomb complexes can potentiate gene expression^133,246,279-282^. For example, a PCGF5-vPRC1 complex that contains AUTS2 and casein kinase 2 and inefficiently ubiquitylates H2AK119, was shown to potentiate transcription by recruiting the histone acetyltransferase P300 (Ref.^279^). Alternatively, the H2AK119ub1-specific reader ZRF1 (also known as DNAJC2) was linked to activation of Polycomb-repressed genes during differentiation^283^. These effects are in agreement with earlier reports of PRC1 supporting gene expression in resting B cells and T cells^280^.

It has also been suggested in some instances that Polycomb chromatin domains can support gene expression by forming chromatin topologies that aid gene induction^57,58,284-286^. Specifically, it has been proposed that, by supporting long-range chromatin interactions, cPRC1 can bring into close proximity enhancers that are poised for activation and their target promoters, thereby enabling rapid induction of transcription in response to activation signals during cell differentiation^58,284,285^ (Figure 6B). This idea is consistent with observations that cPRC1 is associated with enhancer function in breast cancer cells and with activation of genes during cardiac differentiation^59,287^. The precise mechanisms and extent to which Polycomb complexes potentiate gene expression is an exciting area of active investigation.

#### Chromatin bistability and transitions in gene expression

During cellular differentiation, Polycomb target genes can be activated to support new cellular functions^288,289^. This activation is accompanied by loss of the Polycomb chromatin domain and acquisition of an new type of chromatin domain formed through the function of Trithorax group chromatin modifying complexes, which include myeloid/lymphoid or mixed-lineage leukaemia protein 1 (MLL1; also known as KMT2A)–MLL2 (KMT2D) and SETD1A–SETD1B^290^. Interestingly, some Trithorax group complexes can also sample CGIs through zinc finger-CxxC DNA binding domains, but in contrast to the Polycomb complexes, they initiate the formation and spreading of transcription-permissive, H3K4me3-containing Trithorax chromatin domains through communication and feedback mechanisms linked to active transcription^291-296^. Antagonism between the Polycomb and Trithorax complexes helps to ensure that at most gene promoters, either a transcriptionally repressive or a transcriptionally permissive chromatin state predominates.

**Figure 7:**
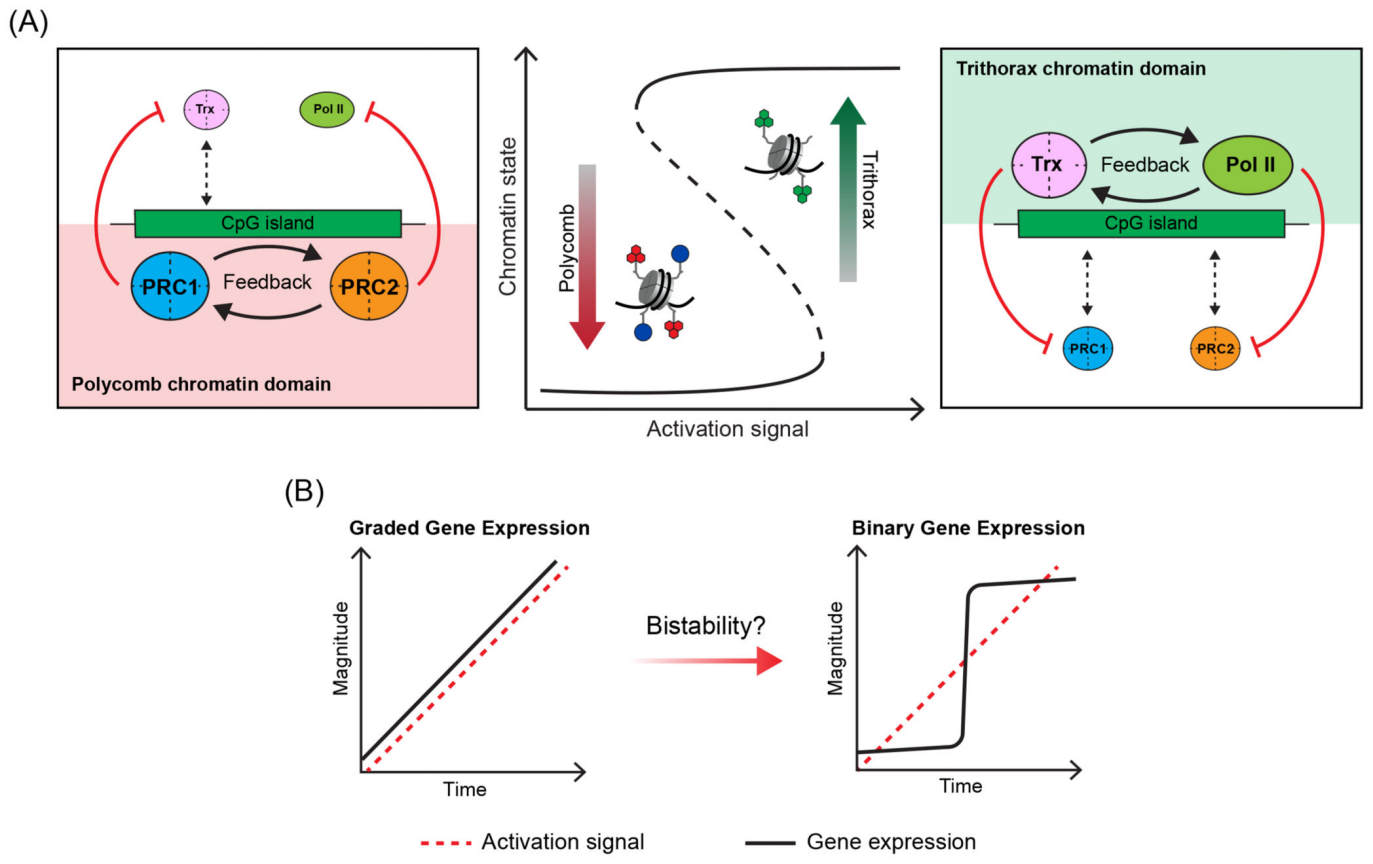
CpG islands and chromatin bistability (A) A schematic illustrating how chromatin bistablity could form at CpG islands (CGIs). When transcription activation signals are low or absent, communication and feedback between Polycomb repressive complex 1 (PRC1) and PRC2 could drive the formation of repressive Polycomb chromatin domains that antagonise Trithorax (Trx) complexes and RNA polymerase II (Pol II) activity (left). When activation signals are high and persistent, communication and feedback between Trithorax complexes and Pol II could drive the formation of transcription-permissive Trithorax chromatin domains that antagonise PRC1 and PRC2 (right). The capacity of both Polycomb and Trithorax systems to sample CGIs coupled with the feedback and antagonistic mechanisms inherent to the formation of each chromatin state, would provide the opportunity to switch between predominantly Polycomb or predominantly Trithorax chromatin states as gene activation signals increase or decrease. We speculate that this mode of gene regulation could help to shape gene expression transitions (see part B) and also provide a chromatin-encoded hysteresis of the current transcriptional state of the gene in the face of inherently stochastic and pulsatile transcription initiation induced from single gene promoters. (B) If transitions between gene expression states, for example gene induction during cellular differentiation, scaled linearly with its activation signal, then one would predict graded expression output (left). However, if CGIs help to create bistable chromatin at gene promoters, this could shape binary, switch-like, gene expression transitions in which Polycomb chromatin domains constrain activation signals until appropriate activation thresholds are reached, at which point transcription initiation would precipitate a rapid switch into a Trithorax chromatin state and potentiate transcription. In the context of such a system, one might predict CGIs could help to convert graded gene activation signals into binary switch-like gene expression outputs through chromatin bistability. This could be particularly useful in supporting decisive gene expression transitions during development. Adapted with permission from REF. 290, Elsevier.

Based on these feedback mechanisms and antagonism between the Polycomb and Trithorax systems, CGIs have been proposed to support the formation of bistable chromatin states at gene promoters to regulate gene expression^99,222,297-300^ (Figure 7A). In defined cell types, the capacity to form either Polycomb or Trithorax chromatin domains could provide memory of gene expression programmes and buffer against low-level or spurious transcription activation signals that could be detrimental to cell identity. However, the convergence of these systems on CGIs might also shape gene expression transitions during differentiation. For example, Polycomb chromatin domains maintained by feedback mechanisms at inactive genes could constrain gene activation signals until an appropriate induction threshold is reached^301^ (Figure 7A). By contrast, following productive initiation of transcription, formation of permissive Trithorax chromatin domains could potentiate transcription while also blocking Polycomb activity. A rapid switch between chromatin states might influence transcription by converting graded gene activation signals into binary gene expression outputs (Figure 7B). In support of this possibility, the Polycomb system has been shown to impart switch-like effects on gene expression in *Arabidopsis thaliana* ^302,303^, and there is some evidence that this gene expression switch also occurs in mammals^304^. We envisage that switch-like transitions in the expression of master regulators of cell fate could help to ensure decisive cell fate decisions, and Polycomb and Trithorax complexes are known to have important roles in regulating these types of genes during mammalian development^3^. Furthermore, once individual genes acquire defined chromatin states, this could provide hysteresis of their current transcription status to help maintain cell identify in the face of inherently stochastic and pulsatile transcription signals.

Recent mathematical models incorporating the known activities of Polycomb and Trithorax systems have provided theoretical evidence to support the existence of bistable chromatin states at gene regulatory elements^297,298,303^. Importantly, interrogation of these models also suggests that bivalent chromatin states, which are characterised by the combined presence of both H3K4me3 and H3K27me3 and previously proposed to define an alternative poised transcriptional state^60,305^, are unlikely to be stable and may instead correspond to the transition between Polycomb and Trithorax chromatin states. Although many of the underlying biochemical activities of Polycomb and Trithorax systems are consistent with the possibility that bistable chromatin states could be created at their target sites, a major challenge for our research field will be to directly test these models and their relevance to the kinetics of gene expression during cell differentiation.

### Conclusion and future perspective

Detailed biochemical and structural characterisation has revealed that the Polycomb system comprises a diverse array of multi-protein complexes that are highly specialised in how they engage with nucleosomes, catalyse histone modifications and function through communication and feedback mechanisms to form Polycomb chromatin domains. Our understanding of the mechanics that underpin these processes has advanced rapidly in recent years, but it still remains poorly understood at the mechanistic level how, once formed, Polycomb chromatin domains influence the transcription machinery to enable gene repression. This represents a central unresolved question in our field. Given the complexity of gene regulatory elements *in vivo*, we anticipate that addressing this important question will require a reductionist approach leveraging *in vitro* transcription reactions on reconstituted chromatin templates, and single-molecule approaches that can capture the behaviour of the transcription machinery^306,307^.

Building on these biochemical underpinnings, the molecular study of how Polycomb systems function *in vivo* has indicated they are more dynamic than originally anticipated and, somewhat counterintuitively, appear to respond to transcription in forming Polycomb chromatin domains. This is forcing us to rethink how the Polycomb system both influences, and is related to, gene transcription. In this Review, we have discussed some emerging molecular principles that could explain these dynamic behaviours. However, the static and ensemble experimental approaches that our field has relied on until now are largely unsuited to exploring the relevance of these concepts. Instead, this will require live, single-cell, and quantitative measurements. These approaches will be of particular relevance in determining whether the Polycomb system enables chromatin bistablity, how it shapes the kinetics of gene-expression transitions during cell differentiation and development, and how the functions of Polycomb complexes are disrupted in human disease.

Finally, much of our understanding of the mammalian Polycomb system has come from studies in stem cells and in early developmental contexts. Although the ideas and models discussed in this Review primarily draw on this body of work, it is becoming increasingly clear that the mechanisms that guide Polycomb function in specific tissues and at later developmental stages are likely to vary considerably. Therefore, moving forward, a central objective remains to uncover the complement of mechanisms and principles that define mammalian Polycomb-mediated gene regulation in diverse cellular and developmental contexts.

As our field ventures into testing the relevance of new and emerging concepts in Polycomb biology, the coming years will inevitably be a fast-paced and exciting period in the ongoing quest to uncover the molecular principles that define gene regulation by Polycomb repressive complexes.
